# Management of pregnancy in women with rare multisystemic vascular diseases: a qualitative survey analysis

**DOI:** 10.1186/s13023-026-04472-6

**Published:** 2026-06-27

**Authors:** Gloria Somalo-Barranco, Alexandra Benachi, Laurence M. Boon, Petra Borgards, Janine Dickinson, Freya Droege, Olivier Dupuis, Michael Frank, Guillaume Jondeau, Robert M. Kauling, Diana Marinello, Edit Nagy, Jolien W. Roos-Hesselink, Meike Rybczynski, Pernille Mathiesen Torring, Carine van der Vleuten, Aline Verstraeten, Liesbeth Wildero Van Wouwe, Dina Zucchi, Julie De Backer

**Affiliations:** 1 Hôpital Bichat, VASCERN Coordination Team, Paris, France; 2 Department of Obstetrics and Gynecology, DMU Santé des Femmes et des Nouveau-nés, Assistance Publique Hôpitaux de Paris, Antoine Beclere Hospital, Université Paris-Saclay, Paris, France; 3 Division of Plastic Surgery, Center for Vascular Anomalies, Saint-Luc University Hospital, UCLouvain, Brussels, Belgium; 4 VASCERN VASCA European Reference Centre, Brussels, Belgium; 5 Bundesverband Angeborene Gefäßfehlbildungen e.V., Federal Association of Congenital Vascular Malformation, Mülheim an der Ruhr, Germany; 6 Department of Dermatology, Phlebology and Lymphology, Nij Smellinghe Hospital, Drachten, The Netherlands; 7 VASCERN PPL European Reference Centre, Drachten, The Netherlands; 8 Department of Otorhinolaryngology, Essen University Hospital, University of Duisburg-Essen, Essen, Germany; 9 VASCERN HHT European Reference Centre, Essen, Germany; 10 Hospices Civils de Lyon, Service de Gynécologie-Obstétrique, Hôpital Lyon-Sud, Pierre-Bénite, France; 11 VASCERN HHT European Reference Centre, Lyon, France; 12 Département de Génétique, Centre de Référence des Maladies Vasculaires Rares and VASCERN MSA European Reference Centre, AP-HP, Hôpital Européen Georges Pompidou, Paris, France; 13 INSERM, U 970, Paris Centre de Recherche Cardiovasculaire-PARCC, Paris, France; 14 Department of Cardiology, Centre de référence pour le syndrome de Marfan et apparentés, AP-HP, Université Paris Cité, Hôpital Bichat-Claude Bernard, Paris, France; 15 VASCERN HTAD European Reference Centre, Paris, France; 16 Department of Cardiology, Erasmus Medical Center, Rotterdam, The Netherlands; 17 VASCERN HTAD European Reference Centre, Rotterdam, The Netherlands; 18 Rheumatology Unit, Azienda Ospedaliero Universitaria Pisana, Pisa, Italy; 19 Division of Valvular and Adult Congenital Heart Disease, Department of Cardiology, Karolinska University Hospital, Theme Heart and Vessels, Stockholm, SE-171 76 Sweden; 20 VASCERN HTAD European Reference Centre, Stockholm, Sweden; 21 University Hospital Hamburg-Eppendorf University Heart Centre, Hamburg, Germany; 22 VASCERN HTAD European Reference Centre, Hamburg, Germany; 23 Department of Clinical Genetics, Odense Universitetshospital, Odense, Denmark; 24 VASCERN HHT European Reference Centre, Odense, Denmark; 25 Department of Dermatology, Radboudumc Expertise Center for Haemangiomas and Congenital Vascular Malformations Nijmegen (Hecovan), Radboud University Medical Center, Nijmegen, The Netherlands; 26 VASCERN VASCA European Reference Centre, Nijmegen, The Netherlands; 27 Center of Medical Genetics, University of Antwerp and Antwerp University Hospital, Antwerp, Belgium; 28 VASCERN HTAD & MSA European Reference Centre, Antwerp, Belgium; 29 Center for Medical Genetics, Ghent University Hospital, Ghent, Belgium; 30 VASCERN HTAD European Reference Centre, Ghent, Belgium; 31 Rheumatology Unit, Department of Clinical and Experimental Medicine, University of Pisa, Pisa, Italy; 32 Department of Cardiology, Ghent University Hospital, Ghent, Belgium

**Keywords:** Pregnancy, Vascular disease, Maternal risk, Foetal risk, European Reference Network

## Abstract

**Background:**

Pregnancy in women with rare vascular diseases is highly challenging, as it can be associated with significant maternal and foetal risks, requiring complex and multidisciplinary management. Care across countries remains insufficiently characterized, and both patients and healthcare providers across Europe highlight the need for better structures and organization to support this critical and potentially life-threatening phase.

**Objective:**

To describe current practices, perspectives, and challenges faced by patients and healthcare professionals in the management of pregnancy in women with rare vascular diseases.

**Methods:**

A survey developed by the European Reference Networks (ERN) pregnancy working group was extended with VASCERN-specific questions, resulting in a 13-item questionnaire. The survey was distributed to healthcare providers and patient representatives (ePAGs). Qualitative data from open-ended responses were analysed using an inductive thematic approach.

**Results:**

36 responses were collected from 145 invited VASCERN members, resulting in a 25% response rate and representing 10 European countries. Most respondents were healthcare providers (83%). 17% were patient representatives. Thematic analysis identified five recurring themes, including the need for coordinated and collaborative multidisciplinary approaches, variability in the practices regarding preconception and genetic counselling, limited access to specialised centres for pregnancy monitoring, and the importance of structured delivery planning and postnatal follow-up.

**Conclusions:**

This survey clearly shows that there are common concerns about pregnancy in women with rare vascular diseases. Improvement actions mainly involve better education and drawing up multidisciplinary care pathways and guidelines that will lead to better access and organisation of care. Initiatives in this direction have already been taken within VASCERN.

## Background

Preparing and managing pregnancy in women with rare vascular diseases poses significant challenges due to potential maternal and foetal complications and risks associated with these conditions.

Both from the perspective of patients (represented by the European Patient Advocacy Group, ePAG) as well as healthcare providers (HCP) across Europe, there is a clear need to improve the structures and organisation to support this crucial event in life, not least due to potential life-threatening complications.

Because many of these concerns are almost universal for all rare diseases, the European Reference Networks (ERNs) created a survey to gauge the current situation and identify unmet needs. In total, 197 answers were collected from HCPs and ePAGs from 24 different countries. Reported unmet needs included a need to improve inter-HCP communication, a lack of predefined organizational pathways, a lack of availability of expert HCPs for some pregnancy-related issues and a need for streamlining care provided among different countries. The survey results also underlined the need to improve the educational activities provided to patients with rare diseases [1].

However, while these findings provide important general insights, disease-specific challenges related to pregnancy in women with rare vascular diseases remain insufficiently characterized. This includes care organization, access to expertise, and cross-country variability within and across European healthcare systems. To address this gap, within the ERN for Rare Vascular Diseases (VASCERN), a transversal pregnancy group has been set up, involving members from the six Rare Disease Working Groups (Hereditary Haemorrhagic Telangiectasia, Hereditary Thoracic Aortic Disease, Medium Sized Arteries, Neurovascular Diseases, Paediatric and Primary Lymphedema and Vascular Anomalies). Regular meetings were scheduled, and the ERN survey was reviewed and extended addressing specific needs for patients with rare vascular diseases. Both care providers and patients were asked to fill in a structured online survey.

In this document, we report the results of the survey within VASCERN. The main purpose of this document is to describe current practices, perspectives, and challenges faced by patients and HCPs in managing pregnancy in women with rare vascular diseases, as well as to identify potential areas for improvement.

## Methods

The questionnaires provided by the transversal ERN pregnancy Working Group (WG) included 10 items that were universal across all ERNs. To address disease-specific aspects relevant to rare vascular conditions, 3 additional questions were developed within the VASCERN pregnancy WG. These questions were formulated through expert discussions involving representatives from all Rare Disease Working Groups (RDWGs) within VASCERN. All working groups existing in VASCERN at the time were represented: Hereditary Haemorrhagic Telangiectasia (HHT), Hereditary Thoracic Aortic Disease (HTAD), Medium Sized Arteries (MSA), Paediatric and Primary Lymphedema (PPL), and Vascular Anomalies (VASCA). A sixth group specialized in rare cerebrovascular diseases (NEUROVASC) was included after the distribution of the survey, and therefore they did not contribute to it. The final questionnaire therefore consisted of the following 13 items, covering key domains of pregnancy care:

1. ERN transversal survey Question topics
  1. Fertility preservation
  2. Pre-conceptional counselling
  3. Pre-implantation diagnosis
  4. Prenatal diagnosis
  5. Family Planning counselling
  6. Pregnancy Monitoring
  7. Delivery
  8. Post pregnancy monitoring
  9. Lactation monitoring/counselling,
  10. New-born management
2. VASCERN-specific questions
  1. Prenatal Foetal Ultrasound
  2. Pre-conceptional clinical evaluation
  3. Pregnancy Monitoring (anaesthesiology/foetal and maternal imaging)

Each question was answered through a scale structure on the one hand and open-ended answers could also be given on the other hand, in order to allow participants to elaborate on their experiences and perspectives.

In 2022, the survey was distributed electronically to VASCERN HCPs and European Patient Advocacy Groups (ePAGs). The survey remained open for a period of 23 weeks, during which respondents were encouraged to provide detailed and descriptive responses. The qualitative data analysis of the survey revealed recurring themes and patterns in respondents’ open-ended responses, providing valuable insights into their perspectives and experiences. Thematic coding and content analysis were employed to extract meaningful narratives and understand the underlying sentiments expressed in the qualitative data.

Given the limited number of responses and heterogeneity across RDWGs, quantitative analysis of the responses collected was considered descriptive only. Therefore, the primary focus of the analysis was on qualitative data derived from open-ended responses, which provided valuable insights into participant’s perspectives and experiences.

Qualitative analysis was conducted using an inductive thematic approach. Six authors independently reviewed and coded the responses to identify patterns and to extract meaningful narratives. Discrepancies were resolved through consensus within the VASCERN Pregnancy WG. Given the relatively small dataset, formal inter-rater reliability metrics were not calculated. However, consistency in coding was ensured through repeated joint review and agreement by the group. Coding was performed manually using structured data tables; no specific software was used for the analysis.

Themes were subsequently grouped into broader categories reflecting key stages of pregnancy care. The final thematic structure was reviewed and validated within the VASCERN pregnancy working group.

## Results

**Fig. 1:**
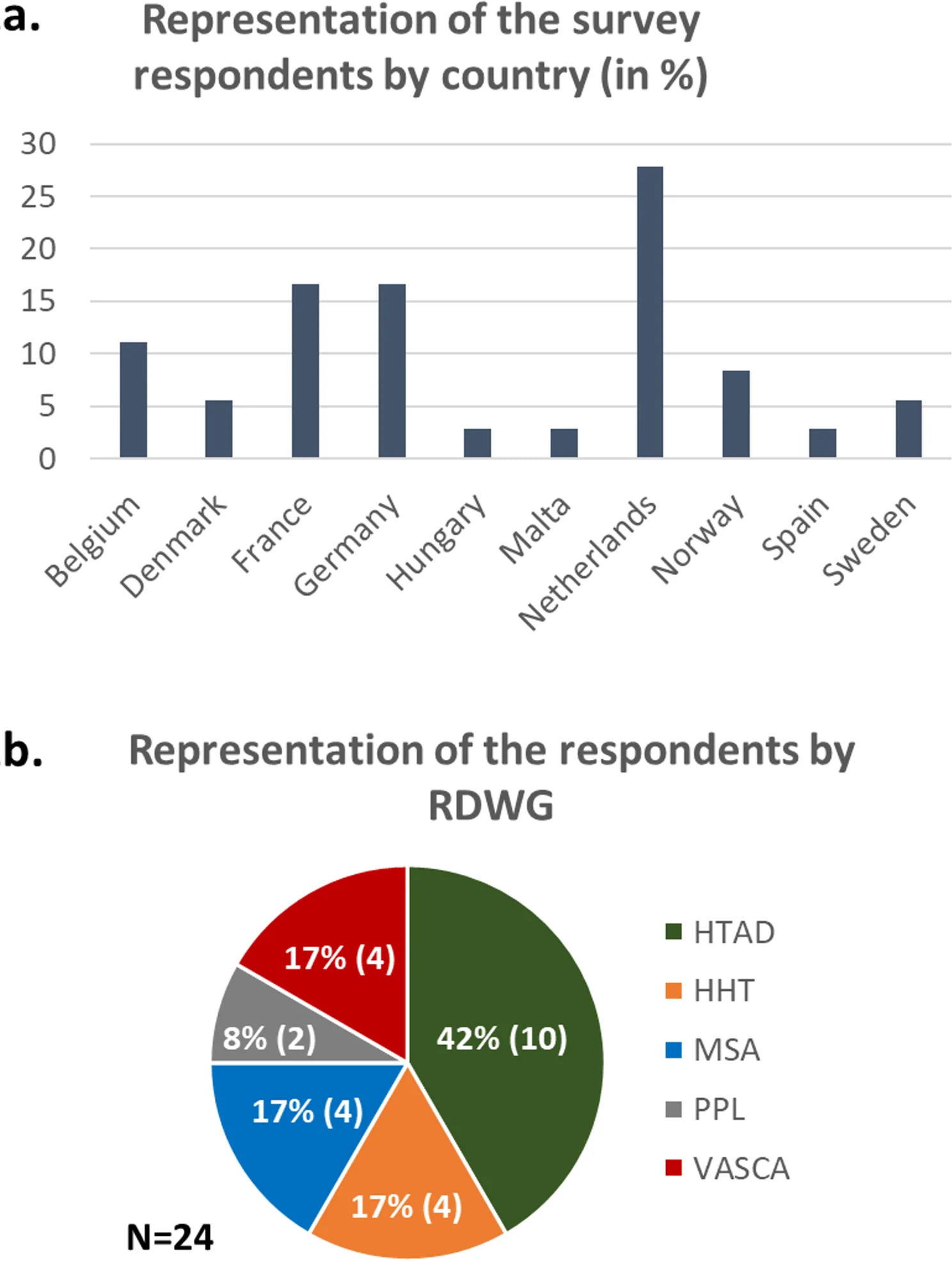
(**a**) Representation of the survey respondents by country: Belgium (4), Denmark (2), France (6), Germany (6), Hungary (1), Malta (1), Netherlands (10), Norway (3), Spain (1) and Sweden (2), out of a total of 36 respondents. (**b**) Representation of the survey respondents according to their Rare Disease Working Group (RDWG), out of a total number of 24 participants who disclosed this information. The distribution of the responses according to the RDWGs and compared to their size was as follows: 10 respondents belong to HTAD (out of 18 HCPs), 4 to HHT (out to 7 HCPs), 4 to MSA (out to 7 HCPs), 2 to PPL (out of 8 HCPs) and 4 to VASCA (out of 14 HCPs). HHT: Hereditary Haemorrhagic Telangiectasia, HTAD: Hereditary Thoracic Aortic Disease, MSA: Medium Sized Arteries, PPL: Paediatric and Primary Lymphedema, VASCA: Vascular Anomalies. Percentages calculated with the total number of participants that disclosed their RDWG (24 out of the total 36 respondents)

In total, 36 responses were collected from 145 invited VASCERN members, which represents a 25% response rate. Among the 36 respondents, 10 countries were represented: Belgium (4), Denmark (2), France (6), Germany (6), Hungary (1), Malta (1), Netherlands (10), Norway (3), Spain (1) and Sweden (2) (as illustrated in Fig. 1a). Only 24 out of the 36 respondents disclosed their rare disease working group (RDWG) affiliation, covering all groups active within VASCERN at the time of the survey. Figure 1b shows the representation of the respondents by RDWG.

Among the participants, 83% were HCPs and 17% were ePAGs.

Table 1 presents the response distribution of the survey questions. The following recurring themes were identified from the thematic analysis of the qualitative data:

1. Need for collaborative approaches and multidisciplinary team involvement
  The analysis revealed a consensus among the respondents regarding the importance of a multidisciplinary team (MDT) approach in managing all stages of pregnancy, including preconception, pregnancy monitoring, delivery and postnatal care. The involvement of obstetricians and other specialists such as cardiologists, vascular surgeons, anaesthesiologists, vascular internists, geneticists, haematologists, oncologists and specialized nurses was considered crucial for optimal care, although this could vary slightly according to the disease. However, challenges related to the organisation of these teams were acknowledged, including difficulties in coordinating multiple specialists, variability in team composition across centres, and limited opportunities for structured communication. These findings suggest that, although MDT care is considered essential, its implementation remains inconsistent in practice.

Items 2–5 pertain to the specific phases of pregnancy.

2. Barriers and differences encountered in practices of family planning and prenatal/preconception counselling
  Prenatal and preconceptual counselling are important aspects of the management of rare genetic conditions in regards of maternal/foetal risks and for the risk of disease transmission. The responses in the questionnaire suggest that absence of a structured collaboration between caregivers and geneticists/counsellors could be a significant obstacle to a timely and adequate/appropriate delivery of information to patients.
  In addition, limited availability and accessibility of prenatal diagnostics (PND) was seen as a potential barrier by several respondents. Long waiting lists for appointments to set up the process of preimplantation genetic testing PGT are perceived as a problem. The lack of knowledge about the potential risk of hormonal treatment in the setting of preimplantation genetic testing (PGT) is an important drawback. Differences between the various countries in the EU were also highlighted by the respondents, regarding particularly the differences in cultural, ethical, healthcare system and legal and regulatory backgrounds which affects the access to preconception (genetic) counselling. In some countries, there are no options for PGT or termination of pregnancy, even if the foetus is carrying a severe disease. Appropriate psychological counselling is mentioned as a requirement for proper functioning of the counselling process.

3. Easy (and close) access to specialized centres for pregnancy monitoring
  Access to specialised care was identified as a key determinant of optimal pregnancy management. Respondents indicated that pregnancy monitoring is most effective when coordinated within expert centres, where multidisciplinary expertise is available. This includes consultations (joint or otherwise) with a disease specialist and an obstetrician can be arranged. An expert centre would be the best setting, but distance from the patients’ place of residence is sometimes cited as a barrier given the need for frequent visits.
  It is mentioned that there are some guidelines for the more frequently occurring rare disorders, but that these are often lacking for the ultra-rare forms. This contributes to variability in clinical practice and highlights the need for more structured and widely accessible care pathways.

4. Planning and managing of delivery
  Good cooperation from the MDT for childbirth is also considered necessary by the respondents. Drawing up a concrete and individualized delivery plan is regarded as an important element. Therefore, there should be a clear guideline in case preterm birth takes place in a non-expert centre. In such cases, distance to the centre for the patient obviously plays an important role in implementing an optimal delivery planning.

5. Tailored postnatal care
  Disease-specific follow-up of mother and child are important and vary from condition to condition. An effective postnatal care includes good cooperation between obstetrics and the disease specialists, in which co-localisation of the different teams plays an important role in some of these cases.

### Patient perspective

Although patient representatives accounted for a smaller proportion of respondents (*n* = 6, 17%), their input highlighted several important aspects of care. In particular, patients emphasized the need for clear and accessible information regarding pregnancy-related risks and contraception, as well as the importance of a multidisciplinary care and collaboration between specialists.

Barriers related to access to expert centres and variability in care across countries were also reflected in patient responses. These included differences in healthcare coverage and reimbursement systems, as well as the impact of geographical distance to specialised centres.

Overall, these perspectives were consistent with those reported by healthcare providers and reinforce the importance of patient-centred communication and equitable access to care.

**Table 1:** Descriptive summary of reported practices and barriers across key domains (*n* = 36)

Theme	Challenge or good practice reported
Pre-conceptional counselling	• Difficulties in accessing expertise / referrals (*n* = 8, 22%) • Need of multidisciplinary setting (*n* = 6, 17%) • Ethical considerations (*n* = 3, 8%)
Pre-implantation diagnosis	• Difficulties in accessing expertise / referrals (*n* = 5, 14%) • Waiting lists (*n* = 3, 8%) • Ethical considerations (*n* = 3, 8%)
Prenatal diagnosis	• Ethical considerations (*n* = 5, 14%) • Not available (*n* = 5, 14%)
Family Planning counselling	• Ethical considerations (*n* = 2, 6%) • Disease-variability (*n* = 2, 6%) • Reimbursement options (*n* = 2, 6%)
Pregnancy monitoring	• Distance to the expert center and the need of multidisciplinary consulations in the same center/day (*n* = 7, 19%) • Lack of guidelines / Knowledge gap (*n* = 2, 6%)
Delivery planning	• Prior preparation of the delivery plan (*n* = 8, 22%) • Need of multidisciplinary setting (*n* = 4, 11%)
Postnatal care	• Need of multidisciplinary setting (*n* = 10, 28%)

## Discussion and recommendations for care

Although the diversity of conditions covered by the VASCERN network might prevent the identification of common needs for improvement, we were nevertheless positively inspired by the overlap in perspectives shared by the respondents regarding pregnancy management. In fact, despite the differences in those diseases, there are common infrastructural requirements and challenges within the health care systems, in terms of structure and organisation of care. This study has allowed us to draw some important outlines and key improvements for optimising care from preconception to postnatal care.

Universal factors that need to be considered include limited awareness and knowledge among HCPs about possible relationship between the rare vascular disease and matters of conception, pregnancy and childbirth, lack of standardized protocols, legal issues, resource constraints, and the need for improved patient education and support systems. The aspect of becoming a rare disease parent needs to be carefully addressed [2]. Not surprisingly, these factors show important overlap with the unmet needs reported in the transversal ERN pregnancy team paper by Zucchi et al. [1], as well as in the broader spectrum of rare diseases [3]. Addressing these challenges may improve the overall management of this complex patient population.

### Multidisciplinary care as central requirement

An overriding need for all patients and throughout the entire process is the need for **MDTs** and coordinated care. Good communication between involved specialists is essential and should be embedded in a team structure. Given the complexity and rarity of the conditions, this is a logical concern whose importance to HCPs and healthcare authorities cannot be overemphasised, and probably best addressed in expert centres, concentrating the medical expertise and the patients.

**Fig. 2:**
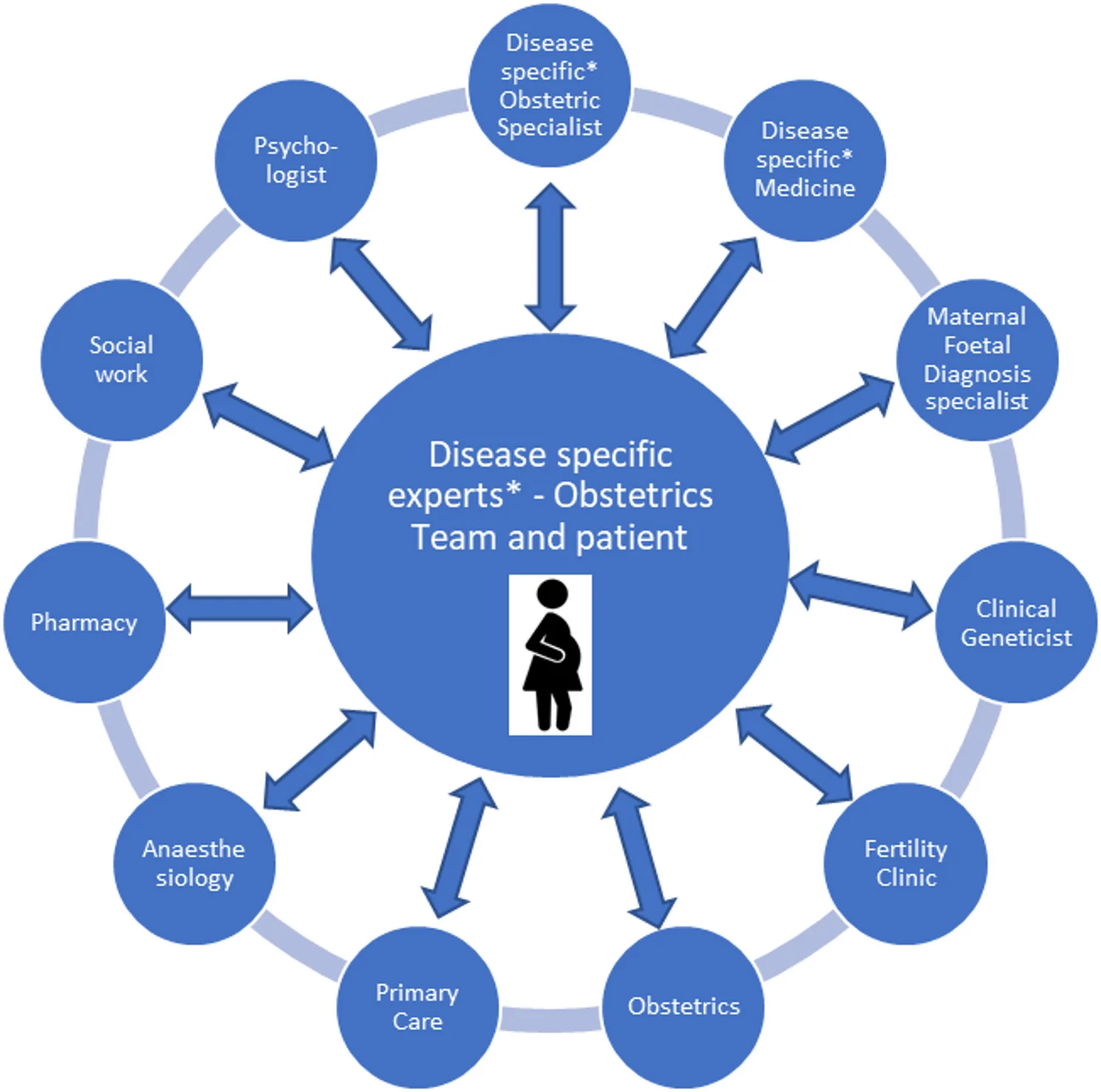
Illustration representing the disease specific obstetrics model of care, in which all specialists collaborate bidirectionally with the obstetrics-patient hub and with each other as clinically required. This model involves a team of specialists working together with the patient to address issues on pregnancy, from preconception, delivery and post-partum care. *Disease specialists will vary according to the underlying rare vascular disease including and not limited to: cardiovascular medicine, haematology, (interventional) radiology, ENT, neurology/ neurosurgery, hepatology

The requirement for MDTs has been adopted in several guideline documents, both in Europe and in the US [4–8]. Requirements will differ according to the disease entities and setting up a program should be tailored to local healthcare legislations. Some guidance is provided in a review document by Easter and colleagues [9]. A generic platform could be envisioned as presented in Fig. 2.

Currently, variable models and pathways of care are in place and little evidence about the (cost) effectiveness of these models is available. A mixed-models evaluation led to the conclusion that evidence-based guidance regarding operationalisation of multidisciplinary care including pathways between local and specialist centres – e.g. for all pregnant women with cardiac disease, for women with extensive vascular malformations affecting the uterus or genitalia, is urgently required [10]. In some specific situations, such as the risk of prematurity, the MDT might face a conflict of interest between the mother and her child when defining the most appropriate level of care for each at delivery.

### Preconception counselling and testing

**Preconception counselling and testing** is a valuable tool that helps individuals and couples make informed decisions regarding their reproductive choices. Although not all elements of preconception counselling apply to all patients with vascular disease, offering the opportunity to discuss child wish and pregnancy, not only with their treating physician but also with a geneticist and/or obstetrician should be given to every couple [11–13]. Preconception counselling should include discussions on contraception and long term-outcome in the patients. Unplanned pregnancies are to be avoided, also taking into account that women may be taking drugs with potential harmful effects. It is crucial to consider various ethical, legal, and practical aspects when implementing such programmes. Ethically, the autonomy and privacy of individuals must be respected, ensuring that they have the right to choose whether to undergo counselling and testing. Informed consent should be obtained, and the potential risks, benefits, and limitations of the tests must be clearly communicated [14]. Information about potential maternal risks related to pregnancy should be given to the patient and spouse when applicable Confidentiality and data protection are vital to maintain trust and safeguard personal information.

From a legal standpoint, there may be considerations regarding the availability, accessibility, and affordability of preconception counselling and testing services [15]. Laws and regulations will vary across countries but should be in place to protect individuals from discrimination based on the results of these tests, ensuring that they do not impact insurance coverage, employment, or other areas of life. Legal frameworks should also govern the storage and use of genetic information to prevent unauthorized access or misuse.

Practically, healthcare systems should be equipped to provide preconception counselling and testing services, addressing clinical risk and – if applicable - genetic aspects of the disease including trained healthcare professionals, appropriate facilities, and necessary (genetic) laboratory capabilities [16]. Ensuring the accuracy and reliability of tests is essential to provide reliable information to individuals and couples. Additionally, the integration of counselling and testing services into existing healthcare infrastructure is crucial to reach a wider population and promote inclusivity [17]. Cross-border programmes and referral should be offered when needed.

Ethical considerations involve respecting individual autonomy and privacy, obtaining informed consent, and maintaining confidentiality. Legal considerations encompass protecting against discrimination, regulating the use of genetic information, and ensuring accessibility. Practical considerations revolve around the availability, accuracy, and integration of preconception counselling and testing services within healthcare systems. Adhering to these considerations helps foster a responsible and inclusive approach to preconception counselling and testing.

### Access to specialized centres for pregnancy monitoring and delivery planning

Once pregnant, appropriate monitoring of the mother and foetus needs to take place – which will again be disease-specific and needs tailoring. Access to expert centres is a key determinant of optimal pregnancy management in complex and rare conditions, as they concentrate expertise and facilitate multidisciplinary collaboration. However, this study highlights that geographical distance and resource constraints may limit access, especially when frequent monitoring is required. As a result, care is often shared between local providers and specialised centres, which may lead to fragmentation if coordination is insufficient.

In addition, while guidelines exist for some more prevalent rare vascular conditions, they are often lacking for ultra-rare diseases. This contributes to variability in clinical practice and underlines the need for more structured and accessible care pathways.

The timely preparation of an **adequate delivery plan** that is discussed with the mother and spouse when applicable and is also accessible to healthcare providers outside the expert centre. This is also especially important in those cases where the distance between the place of residence and the nearest expert centre is substantial. Delivery planning should account for the maternal disease specific status, potential obstetric complications, and the need for specialized care, such as dedicated diagnostic or surgical facilities for the mother and/or neonate [5].

In some rare vascular diseases, active prevention / screening and prompt recognition of complications occurring during pregnancy is required to optimize maternal and foetal outcomes. It has for example been established in many reports that knowledge of the diagnosis of Marfan syndrome was the most important factor ensuring a good maternal and foetal outcome [18–20]. Similar observations apply to other rare vascular diseases such as HHT or other inherited vascular malformations such as Capillary Malformation-Arteriovenous Malformation where recognition of the disorder, followed by recommended screening and treatment, can prevent severe maternal complications [21, 22]. Educating caregivers and patients about the risks and alarm symptoms should be part of the program. Close monitoring throughout pregnancy and the postpartum period is essential [23].

In some cases, adjusting and/or starting **medication during pregnancy** will also be necessary, and good information and communication between healthcare providers and patient is important for this. Medication during delivery and lactation should also be discussed.

Regarding medication in patients with rare vascular disorders, there is still a lot of uncertainty, ranging from feasibility of medication for fertility treatment to effect of certain medication during pregnancy and breastfeeding. To clarify this further, we set up a Delphi study with the VASCERN pregnancy group in order to eventually reach consensus.

### From common challenges to implementation

Despite the heterogeneity of rare vascular diseases, the challenges identified in this study show considerable overlap with previously reported unmet needs within the ERN framework. Importantly, these issues persist even within a coordinated European network, suggesting that the main challenge lies not only in awareness but also in implementation.

Addressing these gaps requires practical measures, including the development of structured care pathways, improved coordination between local and expert centres, and enhanced education for both healthcare providers and patients. Table 2 provides a summary of the recommendations to be implemented to improve pregnancy care in women with rare vascular diseases. Initiatives in this direction have already been initiated within VASCERN and will need to be further developed and adapted to this specific patient population.

### Limitations

This study has several limitations. First, the response rate was relatively low (25%), which introduces a risk of selection bias, as respondents may represent centres with a particular interest or expertise in pregnancy management. Second, the distribution of responses across RDWGs was uneven, limiting the ability to draw conclusions for specific disease groups. Third, patient representatives (ePAGs) accounted for a minority of responses (17%), which may have limited the representation of patient perspectives. Finally, differences in healthcare systems, legal frameworks, and cultural contexts across countries may influence responses and limit the generalizability of the findings.

Given these limitations, the results should be interpreted as exploratory and hypothesis-generating. Nevertheless, the consistency of the themes identified suggests that the challenges described are broadly relevant.

**Table 2:** Summary of the key recommendations for improvement on pregnancy care in women with rare vascular diseases

Elements for improving pregnancy care in women with Rare Vascular Disease			
Domain of care	Action	Implementation of the action	Time-frame (short-term vs. long-term)
Multidisciplinary care	• Define disease specific recommendations for multidisciplinary teams • Evaluate the (cost-) effectiveness of interdisciplinary pregnancy teams	• Local and European level • Local level	• Short-term • Short-term
Access to expertise	• Install strategies to ensure adequate access to appropriate care and referral pathways to expert centres	• Local, national and European level	• Short-term
Preconception	• Offer the entire spectrum of PND/PGT • Develop cross-border programs if needed	• National level • European level	• Long-term • Long-term
Pregnancy	• Harmonize clinical guidance in pregnancy monitoring across European centres where feasible	• European level	• Long-term
Delivery	• Standardize individualized delivery plans	• Local level	• Short-term
Postnatal care	• Establish a structured follow up protocol after delivery adapted to the disease	• Local and European level	• Short-term
Education and training	• Develop and provide educational programs for healthcare providers on cardiovascular-obstetrics • Develop webinars and short videos on rare vascular diseases and pregnancy • Provide accessible patient education tools, available in different languages	• European level • European level • European level	• Long-term • Short-term • Long-term

## Conclusion

This survey clearly shows that there are common concerns about pregnancy in women with rare vascular diseases. On this basis, we can identify some concrete points for improvement, which will mainly involve the development of care pathways, clinical guidelines and specific training programmes that will undoubtedly lead to better organisation of care and better access to specialized expertise. Initiatives in this direction have already been taken within the European expert network, and one of our tasks will be to develop this further and apply it to this specific target group [24, 25].

## Data Availability

The dataset generated and analysed during the current study is not publicly available to keep the answers to open questions confidential but is available from the corresponding author on reasonable request.

## Abbreviations

ERN: European reference network
HCPs: Healthcare providers
HHT: Hereditary Haemorrhagic Telangiectasia
HTAD: Hereditary Thoracic Aortic Disease
MSA: Medium Sized Arteries
PGT: Preimplantation genetic testing
PND: Prenatal diagnostics
PPL: Paediatric and Primary Lymphedema
RDWG: Rare Disease Working group
VASCA: Vascular Anomalies
VASCERN: European Reference Network on Rare Multisystemic Vascular Diseases
